# Atomic-Level Structure Characterization of an Ultrafast Folding Mini-Protein Denatured State

**DOI:** 10.1371/journal.pone.0041301

**Published:** 2012-07-27

**Authors:** Per Rogne, Przemysław Ozdowy, Christian Richter, Krishna Saxena, Harald Schwalbe, Lars T. Kuhn

**Affiliations:** 1 European Neuroscience Institute Göttingen (ENI-G), Göttingen, Germany; 2 DFG Research Center Molecular Physiology of the Brain (CMPB)/EXC 171 “Microscopy at the Nanometer Range”, Göttingen, Germany; 3 Institute for Organic Chemistry and Chemical Biology and Center for Biomolecular Magnetic Resonance (BMRZ), Goethe-University Frankfurt, Frankfurt am Main, Germany; University of Michigan, United States of America

## Abstract

Atomic-level analyses of non-native protein ensembles constitute an important aspect of protein folding studies to reach a more complete understanding of how proteins attain their native form exhibiting biological activity. Previously, formation of hydrophobic clusters in the 6 M urea-denatured state of an ultrafast folding mini-protein known as TC5b from both photo-CIDNP NOE transfer studies and FCS measurements was observed. Here, we elucidate the structural properties of this mini-protein denatured in 6 M urea performing ^15^N NMR relaxation studies together with a thorough NOE analysis. Even though our results demonstrate that no elements of secondary structure persist in the denatured state, the heterogeneous distribution of *R*_2_ rate constants together with observing pronounced heteronuclear NOEs along the peptide backbone reveals specific regions of urea-denatured TC5b exhibiting a high degree of structural rigidity more frequently observed for native proteins. The data are complemented with studies on two TC5b point mutants to verify the importance of hydrophobic interactions for fast folding. Our results corroborate earlier findings of a hydrophobic cluster present in urea-denatured TC5b comprising both native and non-native contacts underscoring their importance for ultra rapid folding. The data assist in finding ways of interpreting the effects of pre-existing native and/or non-native interactions on the ultrafast folding of proteins; a fact, which might have to be considered when defining the starting conditions for molecular dynamics simulation studies of protein folding.

## Introduction

The elucidation of protein folding pathways is inevitably linked to the structural analysis of all experimentally tractable conformations a polypeptide chain can adopt on its path to the final folded form including folding intermediates that represent temporary or local free energy minima along the reaction coordinate [1], [2]. Traditionally, most spectroscopic studies concentrated on either the native state or partially disrupted conformers observed at equilibrium [3]. The structural analysis of the denatured state is considerably more challenging [4]. Despite the apparent difficulty to extract atomic-level experimental information from structurally ill-defined protein states, more recent studies have unambiguously established that residual secondary and/or tertiary interactions can be observed even at high concentrations of denaturant. Moreover, it has been acknowledged that these pre-existing structural elements have to be considered for a more complete understanding of the protein folding process [5]–[8].

Here, we characterize residual structure in the 6 M urea-denatured state of a protein-like peptide known as TC5b [9] which – due to its small size, the limited conformational flexibility, and the ultrafast folding kinetics – has served as an important model for the computational characterization of entire protein folding trajectories using molecular dynamics (MD) simulation studies in the recent past [10]–[18].

**Figure 1:**
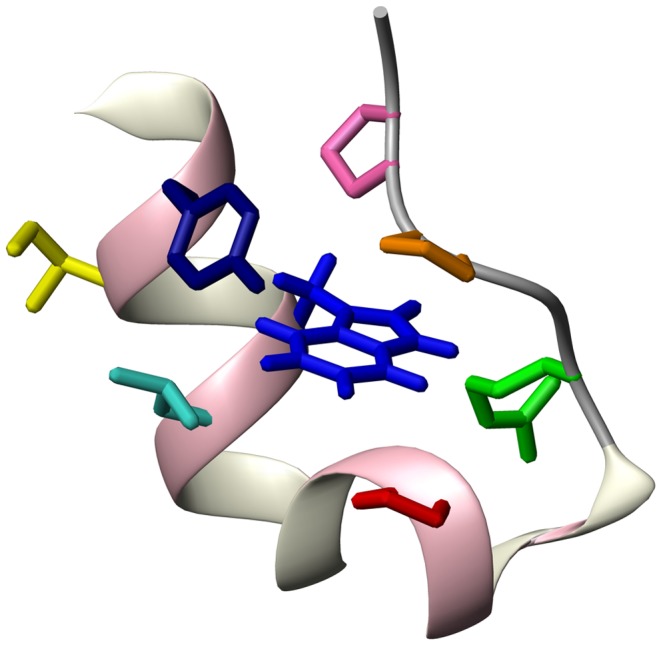
NMR-derived native state structure of TC5b (PDB entry: 1L2Y). Highlighted is the Trp 6 residue (blue) (see text) together with the cage-forming side chains of Tyr 3 (dark blue), Ile 4 (yellow), Leu 7 (light blue), Pro 12 (red), Arg 16 (green), Pro 18 (brown), and Pro 19 (pink).

The previously reported, high resolution NMR structure (PDB entry: 1L2Y, Figure 1) of TC5b’s (NLYIQ WLKDG GPSSG RPPPS) native state reveals a compact hydrophobic core where three proline residues – namely Pro 12, Pro 18, and Pro 19– and a glycine residue (Gly 11) pack against the aromatic side chains of Tyr 3 and, in particular, Trp 6 which lies in the centre of the hydrophobic cage, well shielded from solvent exposure [9]. Secondary structure elements present in the native state comprise an alpha-helix between residues 2 and 8 together with a short 3_10_-helix spanning residues 11 to 14. Vibrational circular dichroism (VCD) studies further demonstrate the presence of a polyproline II helix (PPII) found at the C-terminal end of the peptide [19]. The globular fold of TC5b is structurally maintained by intramolecular hydrogen bonds and a salt bridge between Asp 9 and Arg 16. In addition, the arginine side chain further interacts with the hydrophobic core of TC5b leading to increased fold stabilization of the native state [9], [20], [21]. Due to their highly compact and globular character, TC5b and several other systems derived from this tryptophan-cage motif constitute the smallest fully folded peptides known to date [22], containing not only features of secondary structure but also elements of pronounced tertiary interactions and a protein-like folding behavior [23]. Kinetic investigations into TC5b’s folding reveal ultrafast folding characteristics – despite its high relative contact order – with refolding rate constants on the order of about 4 per microsecond or even below [24], [25]. Moreover, CD and fluorescence spectroscopy experiments demonstrate that folding of TC5b is largely cooperative following a transition of two structurally discrete and hence experimentally accessible states [9], [26].

Early on, it was found that the original TC5b sequence represents an ideal model system for MD folding studies due to its small size, the limited conformational flexibility, and the fast folding [10]. Hence, a plethora of different computational methods employing various boundary conditions and force fields have been tested in recent years to determine the overall folding trajectories and conformational changes that occur during folding [11]–[18]. The methods employed range from *ab initio* calculations carried out *in vacuo* or in the presence of implicit solvation shells [11], [12] to more sophisticated experiments performed in explicit solvent [13] using sometimes more recently established force fields and/or newly designed algorithms [14], [16]. Remarkably, final structures obtained from many simulation studies closely resemble the native NMR-derived TC5b structure, with C_α_ RMSD values as low as 0.96 Å [12]. Nonetheless, most of these calculations started with a fully extended ensemble of TC5b molecules and hence, the folding trajectories observed are likely to be significantly different from those encountered in spectroscopic experiments. Among the numerous computational attempts published, a few results seem to suggest that non-random residual interactions are possibly formed either in the protein’s denatured state or, alternatively, during folding. Calculations carried out by Zhou, for example, using a force field with periodic boundary conditions in explicit water support the experimental observation of early hydrophobic clustering in non-native states of TC5b [13]. Another study which employed short, i.e. 30 ns, stochastic dynamics simulations used a fully relaxed unfolded ensemble with a C_α_ RMSD of 4.8 Å as starting state [11]. In this investigation, a transient non-native intermediate was observed during a representative folding trajectory, suggesting that even for such a small protein, non-native as well as native interactions can be formed along the folding pathway. Hence, both studies supported the observation of pre-existing structure in non-native ensembles of TC5b and its importance in the ultrafast folding of the mini-protein. The somewhat contrary results of most other simulation studies, however, did not take the presence of residual interactions in unfolded ensembles of TC5b into account.

In contrast to the wealth of computational data dealing with the folding behaviour of TC5b, only very little experimental findings concerned with the structural characteristics of the denatured state are presently available: initial NMR experiments conducted on the temperature-denatured state performed by Andersen and co-workers suggested that this conformation of the peptide does not display random coil ^1^H chemical shift values for all of the side chain protons but rather exhibits significant chemical shift deviations (CSDs) for some ^1^H nuclei more characteristic for the presence of persistent non-random interactions [9]. This observation was further confirmed by independently performed fluorescence correlation spectroscopic (FCS) experiments conducted by Neuweiler *et al.* [27] and by photo-chemically induced dynamic nuclear polarization (CIDNP) NMR NOE transfer studies by Hore and co-workers [28]. Interestingly, the CIDNP study provided residue-specific data showing that prior to folding the TC5b molecule exists in a hydrophobically collapsed state comprising both native as well as non-native contact interactions between Trp 6 and several other, mostly aliphatic, side chains, e.g. Ile 4, Leu 7, Pro 12, and Arg 16.

In the present contribution, these structural studies on 6 M urea-denatured TC5b are extended providing residue-specific ^13^C chemical shifts and a NOE analysis together with heteronuclear (^15^N) relaxation studies of the amino acid backbone using uniformly ^13^C- and ^15^N-labelled peptide. These data, combined with structural and dynamic information on two TC5b point mutants, serve to further elucidate the structural origin of the ultrafast folding kinetics of this class of mini-proteins.

## Results and Discussion

### NMR Characterization of Native TC5b

**Figure 2:**
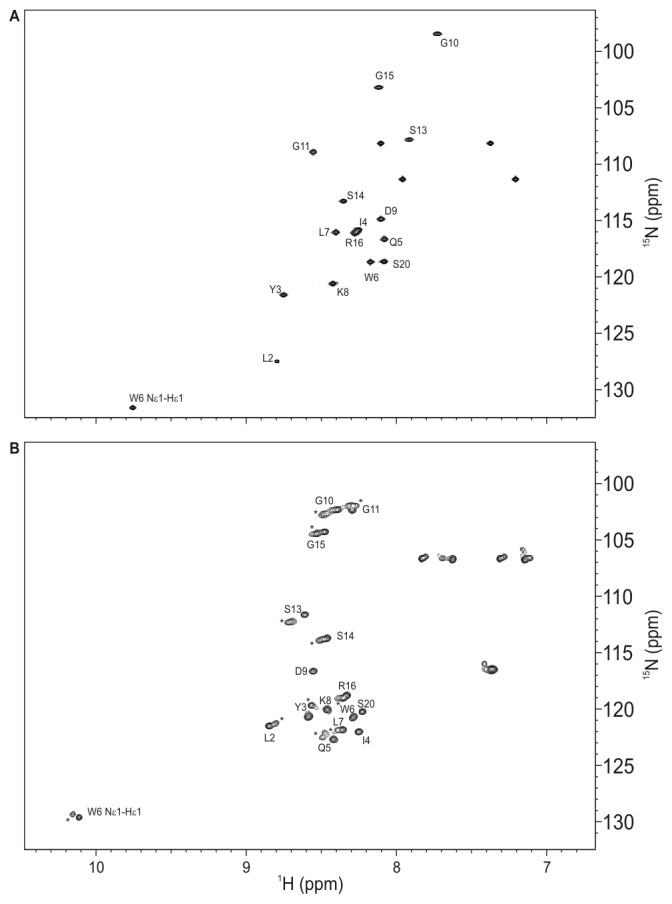
^1^H-^15^N HSQC 600 MHz NMR spectra of TC5b. Shown are native (upper part) and 6 M urea-denatured (lower part) spectra acquired in aqueous buffer solution (25 mM sodium phosphate, native: pH 6.8, 278 K; denatured: pH 3.5, 298 K). Signals representing the backbone NH nuclei and the Trp 6 indole NH unit are labelled using the one-letter code for amino acids. NH cross-peaks stemming from the small fraction of *cis*-Pro isomers in the case of 6 M urea-denatured TC5b are marked with an asterisk.

We first performed a NMR resonance assignment utilizing heteronuclear ^15^N NMR data of native and the 6 M urea-denatured states of TC5b. Figure 2A shows the ^1^H-^15^N HSQC (heteronuclear single quantum coherence) spectrum of native TC5b. The 2D correlation spectrum contains the number of backbone ^15^NH cross-peaks indicative of a single form of the protein exhibiting well-defined and sharp signals. Moreover, the relatively large chemical shift dispersion of NH backbone cross-peaks, in particular in the ^1^H dimension of the spectrum between 7.65 and 8.8 ppm, underscores the aforementioned protein-like character of TC5b’s native state structure. From ^3^ *J*(H^N^,H_α_) scalar coupling constants, measured in a 3D HNHA pulse experiment [29], the intervening dihedral backbone torsion angle ‘phi’ (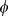) was determined (Table S1). In agreement with circular dichroism (CD) measurements and the NOE cross-peak analysis [9], the determination of backbone torsion angles provides further evidence for the presence of an alpha-helix at the N-terminus comprising the first eight residues of the amino acid sequence and for the short 3_10_-helix located between residues 11 and 14.

### Characterization of Urea-denatured TC5b

The ^1^H-^15^N HSQC correlation spectrum of TC5b acquired in the presence of 6 M urea is shown in Figure 2B. Consistent with fluorescence excitation data and initial NMR measurements [28], the data indicate that the molecule is significantly denatured at high concentrations of urea, as suggested by the significantly reduced chemical shift dispersion of main chain ^15^NH cross-peaks that resonate between 8.2 and approximately 8.8 ppm in the ^1^H dimension. In addition, the peaks are exchange-broadened. The HSQC spectrum of urea-denatured TC5b exhibits a second set of cross-peaks for some of the residues; a fact, which can be attributed to the presence of a minor fraction (20% ±5%) of Pro 12 *cis*-isomers present under denaturing conditions [28].

Despite the reduced spectral dispersion, a complete assignment of both the protein backbone as well as the side chain ^1^H nuclei was achieved following the analysis of two-dimensional proton-proton NMR data: the assigned cross-peaks include 20 H_α_ and 25 H_β_ resonances, a number identical with that achieved for the native protein.

**Figure 3:**
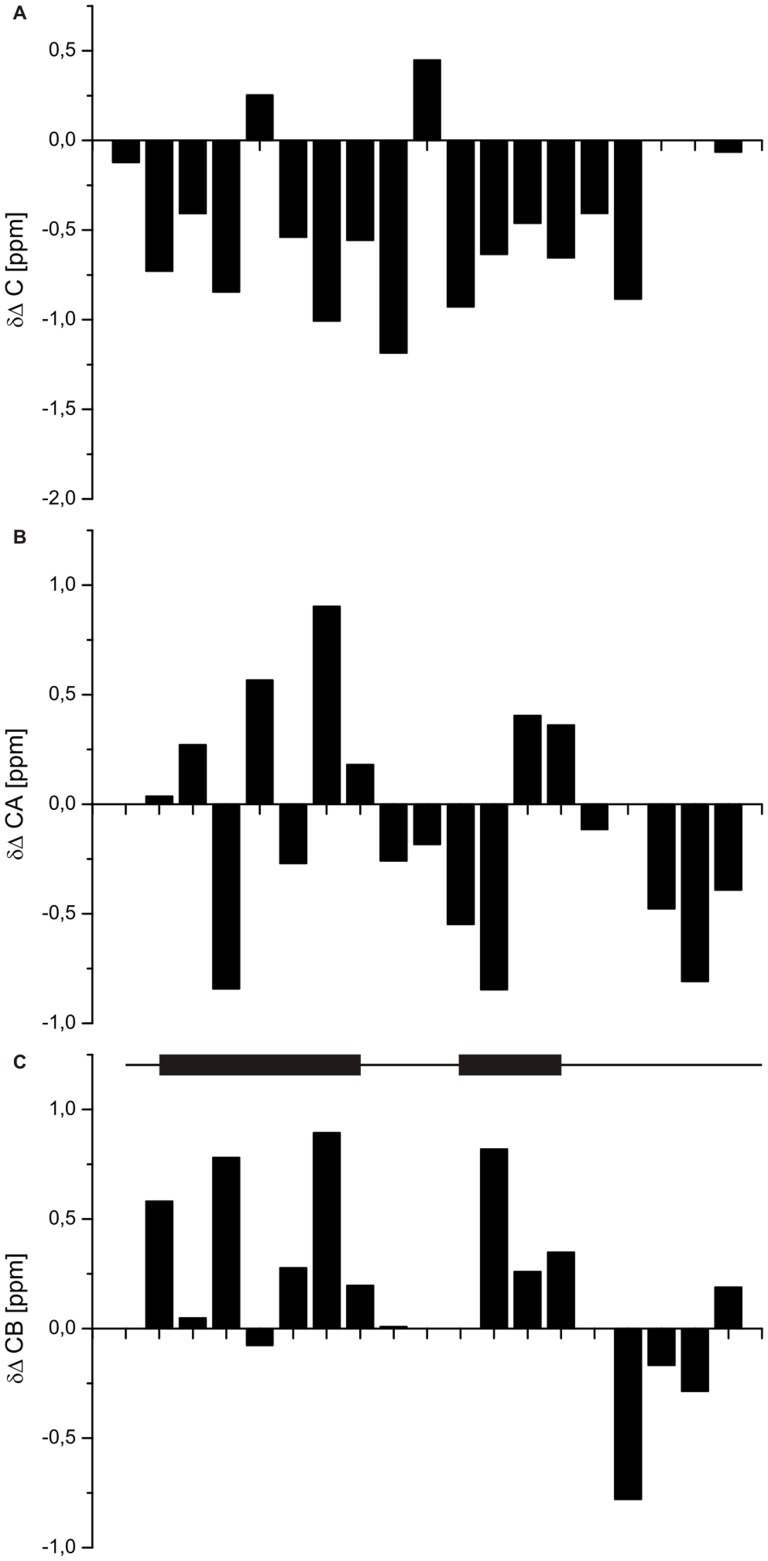
Conformation-dependent ^13^C chemical shifts of resonances of 6 M urea-unfolded TC5b. The Δδ values for the (**A**) C′, (**B**) C_α_ and (**C**) C_β_ resonances are the experimentally determined chemical shifts minus the sequence-corrected random coil values reported by Schwarzinger *et al.* [31]. The positions of the helices in the native protein are indicated in (**C**).

The consensus chemical shift index [30] for the C_α_, C_β_ and C′ resonances of urea-unfolded TC5b using sequence-corrected primary chemical shifts measured in 8 M urea [31] shows that no regular secondary structure element persists in the unfolded state (Figure 3 & Figure S1); an observation further verified measuring ^3^ *J*(H^N^,H_α_) coupling constants (Table S1). Nonetheless, a number of resonances have chemical shifts substantially different from their sequence-corrected primary values. Those with chemical shift differences greater than 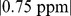 unassociated with the pH difference between the reference chemical shifts and those of 6 M urea-unfolded TC5b reported here are: C_α_, Ile 4, Leu 7, Pro 12,and Pro 18; C_β_, Ile 4, Leu 7, Pro 12, and Arg 16; and C′, Ile 4, Leu 7, Asp 9, Gly 11, and Arg 16. Hence, the chemical shift data alone suggest the presence of significant non-random structure in the urea-denatured state, consistent with observations made for other proteins denatured in urea [7], [32]. Interestingly, some residues exhibiting relatively large chemical shift deviations, have also been identified to participate in the formation of a hydrophobic cluster exploiting data derived from the aforementioned NMR CIDNP studies [28], in particular Ile 4, Leu 7, Pro 12, and Arg 16. A more exact identification of these interactions on a residue-specific level, however, requires the incorporation of proton-proton NOE contact information into the analysis.

### NOE Spectroscopy (NOESY) Connectivities for Urea-unfolded TC5b

**Figure 4:**
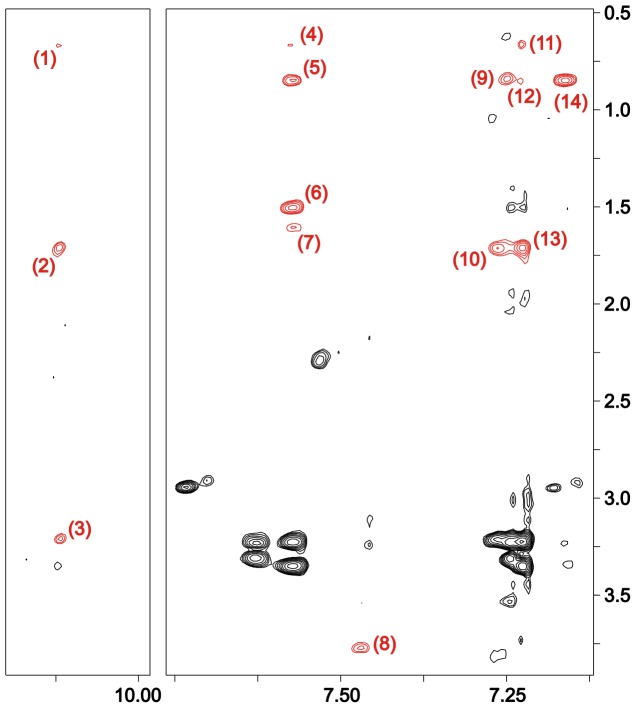
^1^H-^1^H NOESY spectrum of 6 M urea-denatured TC5b acquired at 600 MHz. Shown is the region of the spectrum highlighting sequential and, more importantly, non-sequential NOE cross-peak contacts between aromatic Trp 6 side chain protons and 1H nuclei of several aliphatic side chains (red). Both axes are labeled with the ^1^H chemical shift (ppm). The numbering scheme of the cross-peaks corresponds to the respective side chain contacts as shown in Table 2. Note that cross-peaks indicative of contacts involving H^N^, H_α_, and/or Trp 6 H_β_ protons cannot be seen in this region of the spectrum.

Table 1 provides an overview of all unambiguously assigned inter-proton NOE cross-peaks detected in the 3D ^1^H-^1^H-^15^N NOESY-HSQC- and the homo nuclear NOESY-spectra of 6 M urea-denatured TC5b involving either H^N^ and/or H_α_ nuclei. As can be seen in this representation, numerous sequential H^N^-H^N^ NOEs – black squares in Table 1– were observed despite the limited chemical shift dispersion in the ^1^H dimension; H^N^ to H^N^ 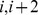 connectivities, characteristic of the presence of helical structure or turns, were, however, entirely absent. In addition, a few NOE cross-peaks between resonances of residue 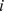 and the H^N^ resonances of residues 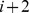 or 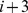 of the peptide backbone were also observed, including a H^N^-H_α_ 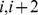 contact between Ile 4 and Trp 6 and two H^N^-H_α_ 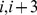 connectivities. The latter are characteristic of helices, but those observed for 6 M urea-denatured TC5b were not numerous enough to suggest the existence of a persistent helical conformation. Thus, the NOESY analysis is consistent with the aforementioned chemical shift data, indicating that no – or at least no significant – residual helical structure persists in 6 M urea-denatured TC5b. The NOEs involving side chain residues demonstrate (Figure 4 & Table 2), on the other hand, that neighbouring and, more importantly, also non-sequential residues in some regions of the sequence interact with each other. This, in turn, would be consistent with the formation of a hydrophobic cluster seen in numerous other temperature- or urea-denatured proteins before [7], [32]–[34]. A closer examination of the observed non-sequential side chain NOE cross-peaks depicted in Table 2 reveals that both the H_β_ as well as all aromatic side chain ^1^H nuclei of Trp 6 interact with several aliphatic residues, i.e. Ile 4 (H_δ1_, H_γ2_), Pro 12 (H_δ2_, H_δ3_), Arg 16 (H_δ_, H_γ_) and the H_δ2/3_ protons of Pro 18 and Pro 19, respectively (Table 2). Furthermore, sequential NOEs of Trp 6 to the two neighbouring residues Gln 5 (H_β_) and Leu 7 (H_β_, H_δ2_, H_γ_) can be detected as well. The NOE cross-peak analysis hence gives an atomic-level insight into the interactions responsible for the formation of a hydrophobic cluster present in the chemically denatured state of TC5b comprising the residues Ile 4, Gln 5, Leu 7, Pro 12, Arg 16, and Trp 6, located at the cluster’s centre. Interestingly, all these interactions do not only involve contacts also present in the native state of TC5b but also comprise additional non-native interactions between the aromatic and aliphatic protons of the tryptophan, on the one hand, and the side chain of Ile 4. These two residues, however, do not exhibit a single NOE contact with each other in TC5b’s native state; in fact, the two side chains can be found on opposite sides of the N-terminal alpha-helix present in the native state of the peptide (Figure 1). Thus, this finding complies with previous observations of native Trp 6/Leu 7, Trp 6/Pro 12, and Trp 6/Arg 16 contacts and non-native Ile 4/Trp 6 interactions. In addition, the Trp 6/Pro 12 contact interaction described in this analysis has also been mentioned by Neidigh *et al.* who interpreted large CSDs for the H_δ3_ proton of the Pro 12 side chain being caused by its interaction with Trp’s aromatic ring current. The presence of the cluster can be further verified through the identification of several long-range NOEs involving cluster-forming side chains other than Trp 6; e.g. a readily identifiable cross-peak between the H_δ2_ proton of Pro 12 and the H_γ_ proton of Arg 16 in addition to a cage-spanning Ile 4 (H_γ2_)/Leu 7 (H_β_) NOE contact. On the other hand, proton-proton NOEs between the side chain nuclei of Asp 9 and Arg 16– separated, on average, by approximately 4.5 Å in the NMR structure of the native state – cannot be detected. Despite its crucial role in stabilizing the globular fold of the native peptide [9], [20], [35], the denatured state hence seems to lack the Asp 9/Arg 16 salt bridge, and the results presented here seem to suggest that it is rather the aliphatic part of the arginine side chain found to be associated with Trp 6 in the 6 M urea-denatured state.

**Table 1:** Assigned main chain contacts between H^N^ and/or H_α_ nuclei.

	N	L	Y	I	Q	W	L	K	D	G	G	P	S	S	G	R	P	P	P	S
*i*, *i*+1	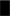	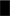	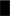	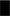	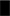	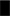	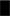	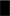				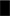	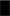							
*i*, *i*+2	Δ			Δ	Δ	Δ	Δ					Δ								
				▴	▴									▴						
*i*, *i*+3				•	•															

Assigned main chain NOE cross-peaks involving either H^N^ and/or H_α_ nuclei for 6 M urea-denatured TC5b. Residues found in the helical regions of the native mini-protein are shown in bold. An NOE between the H^N^ proton of residue 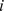 and the H^N^ proton of residue 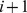 is marked as a black square, NOEs between the H_α_ of residue 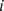 and the H^N^ of 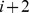 as a filled triangle, and an NOE between any atom of the side chain of residue 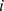 and the H^N^ of residue 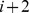 as an open triangle. NOEs between any atom of the side chain of residue 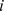 and the H^N^ of residue 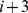 are shown as a black circle.

**Table 2:** Experimentally verified inter-residue contacts between Trp 6 and aliphatic side chains.

	Ile 4	Gln 5	Leu 7	Pro 12	Arg 16	Pro 18	Pro 19
**H_β2/3_**		H_β_	H_β_			H_δ3_	H_δ2_
**H_δ1_**	H_γ2_ (11)		H_δ2_ (12)		H_γ_ (13)		
**H_ε1/3_**	H_δ1_ (5)/H_γ2_ (1,4)		H_β_ (6)/H_δ2_ (5)/H_γ_ (7)		H_δ_ (3)/H_γ_ (2)		
**H_η2_**	H_δ1_ (9)		H_δ2_ (9)		H_γ_ (10)		
**H_ζ2/3_**	H_δ1_ (14)		H_δ2_ (14)	H_δ2_ (8)/H_δ3_ (8)			

1 H-^1^H NOE cross-peaks of the TC5b all-*trans* Pro isomer denatured in 6 M urea. Shown are all detected and assigned sequential and non-sequential NOE contacts between side chain ^1^H nuclei of Trp 6 (bold) and protons of those side chains found to be associated with the tryptophan residue in the chemically denatured state; contacts involving H^N^, H_α_, and/or Trp H_β_ protons cannot be seen in this region of the spectrum. The numbers shown in brackets correspond to the assignment scheme of the respective NOE cross-peak as shown in Figure 4.

### Polypeptide Chain Dynamics of Urea-unfolded TC5b

**Figure 5:**
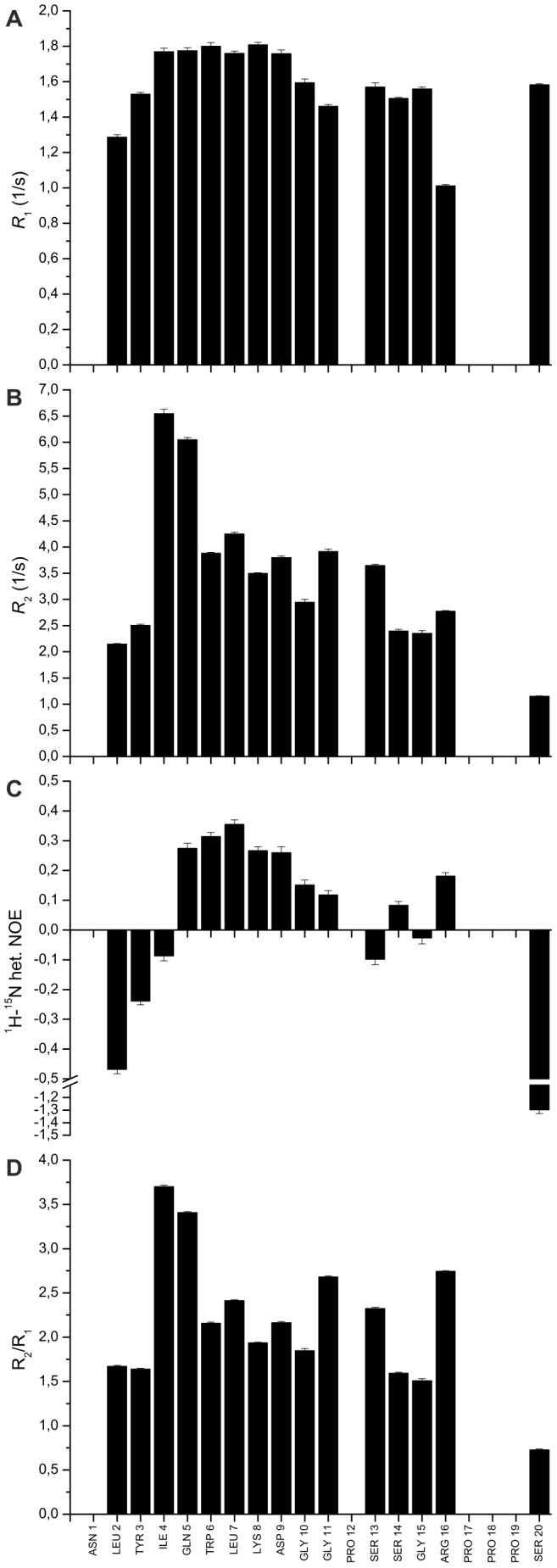
^15^N Relaxation Data. Backbone 60.25 MHz ^15^N relaxation rates and ^1^H-^15^N heteronuclear NOEs of 6 M urea-denatured TC5b acquired with 25 mM sodium phosphate buffer, pH 3.5, and at 298 K. (**A**) *R*_1_ relaxation rates; (**B**) *R*_2_ relaxation times; (**C**) ^1^H-^15^N heteronuclear NOEs; (**D**) R_2_/R_1_ ratios.

To evaluate the structural implications of the NOE contacts discussed in the previous paragraph, peptide backbone dynamics on the high picosecond to low nanosecond timescale of 6 M urea-denatured TC5b were investigated by measuring ^15^N *R*_1_, ^15^N *R*_2_ relaxation rates, and, in addition, ^1^H-^15^N heteronuclear NOE values recorded at a ^15^N frequency of 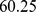 MHz. Relaxation rates were determined for 15 out of the 20 residues, with *R*_2_ values ranging from 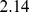 to 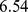 s^−1^, with an average of 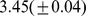 s^−1^ and the average *R*_1_ value being 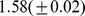 s^−1^ (Figure 5). The ^1^H-^15^N NOEs also showed a wide variation, i.e. from 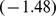 to 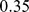. As such, an overall interpretation of the data indicates flexibility throughout the entire sequence of denatured TC5b, as demonstrated most clearly by the ^1^H-^15^N heteronuclear NOEs, which, on average, are considerably lower than ca. 0.75; a typical value expected for backbone amide groups of a natively folded protein [36]. The spin-lattice relaxation rate (*R*_1_) data does not serve to identify regions of restricted backbone motion unambiguously due to the rather uniform distribution of *R*_1_ values along the peptide sequence [37]. A sequence-specific analysis of the *R*_2_ data (Figure 5), on the other hand, seems to suggest the presence of significant non-random structure in 6 M urea-denatured TC5b involving a distinct cluster of hydrophobic residues found in regions of the sequence that exhibit helical elements of sec. structure in the native protein. In addition, more elevated values – close to or slightly above 0.45– for the heteronuclear NOE were determined for individual amino acid residues found to exhibit pronounced NOE contacts with aromatic tryptophan protons as well, e.g. the side chains of Ile 4 , Gln 5 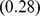, Leu 7 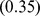, and Arg 16 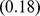 (Table 1). Amino acids closer to the proline-rich ‘tail’ as well as side chains found at the N-terminus of the peptide, on the other hand, show significantly smaller – and in some cases even negative – values for the heteronuclear NOE between 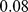 and 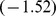. In summary, the residue-specific analysis of both the *R*_2_ and heteronuclear NOE data suggests that transiently populated peptide conformations are more stabilized in the Trp-containing region of the molecule relative, for example, to the C- and N-terminal sections. Hydrophobic clusters of a similar kind have been shown to exist in several other urea-denatured or intrinsically disordered proteins as well [7], [32], [34], [38], [39].

**Figure 6:**
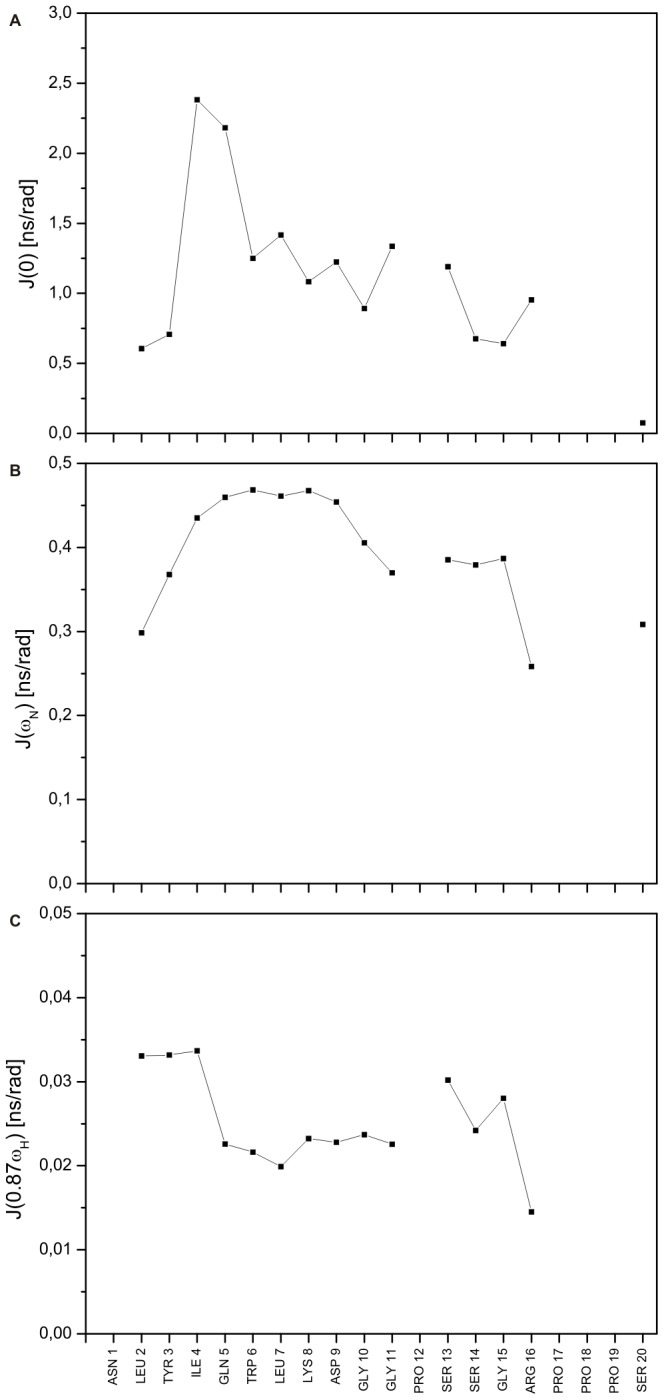
Reduced spectral density functions. (**A**) 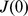, (**B**) 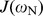 and (**C**) 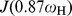 for 6 M urea-denatured TC5b derived from the 60.25 MHz *R*_1_, *R*_2_, and heteronuclear NOE relaxation data from Figure 3. The average errors are 2.5%, 1.2% and 2.7%, for 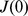, 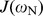 and 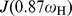, respectively.

Subsequently, the aforementioned relaxation data obtained for 6 M urea-unfolded TC5b was interpreted in terms of the reduced spectral density method [37], [40]–[44] yielding the sequence-dependent variation of the spectral density 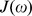 which characterizes the motion of the ^1^H-^15^N bond vector. This approach permits the direct evaluation of 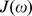 at the three frequencies 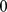, 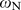, and 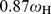 (Figure 6). A close examination of the results shows that 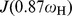 is relatively uninformative regarding the structural interpretation of the backbone dynamics of 6 M urea-denatured TC5b. The analysis of 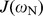 and, in particular, 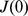, on the other hand, is much more revealing as it shows large variations along the polypeptide sequence thereby reflecting the inhomogeneous distribution of the above-mentioned *R*_2_ data: 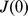 is on the order of 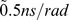 or below at the two termini of the protein and between residues 4 and 16 varies in the range between 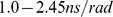. An analysis of the sequence variation of 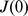 comprising these residues reveals that there is a distinct region comprising residues 4 and 5 having a maximum value of 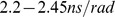 followed by a region exhibiting values on the order of 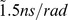. A similar sequence-dependent behaviour of 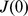 has been observed for many chemically denatured or intrinsically unfolded proteins as well, e.g. the bacterial inhibitor protein Im7 denatured in the presence of 6 M urea, with 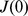 varying in the range 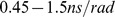 [39] and the natively unfolded colicin E9 translocation domain in the absence of urea, for which 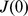 varied in the range between 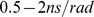 [34]. In all cases, the regions of increased 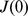 were ascribed to the formation of clusters of interacting residues – mostly non-polar in nature – that constrained the motions of the protein backbone. The correspondence in the magnitude of 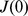 for this cluster suggests that the motional constraints observed in the present case are intrinsic to the type of cluster formed and are not dependent on, for example, urea modulating the cluster dynamics. As such, the data described in this part as well as previously reported relaxation-based NMR structural studies on proteins known to be fully unfolded reveal that the interpretation of the *R*_2_ relaxation rates – taken together with the observation of direct NOE contact interactions between several aliphatic residues (*vide supra*) – provides the most convincing evidence for the presence (or absence) of non-random interactions in intrinsically unfolded or, as in the present case, chemically denatured biomacromolecules [37], [38], [45].

### Order in 6 M Urea-unfolded TC5b

**Figure 7:**
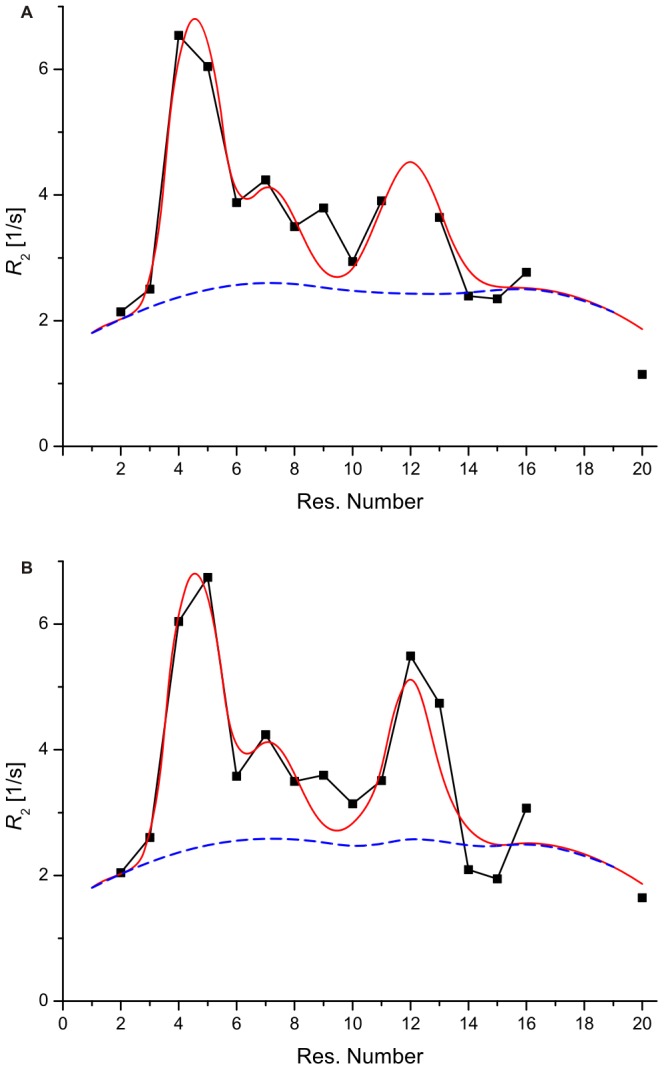
Cluster analysis of denatured TC5b. Plot of the experimental ^15^N *R*_2_ values (black line) and the results of the cluster analysis as described in the text (red line) against the residue number for 6 M urea-unfolded (**A**) TC5b and (**B**) its P12W mutant. Moreover, the dashed line (blue) represents the intrinsic ^15^N *R*_2_ relaxation rates of the polypeptide as described by the first term of Eqn. 1.


Further insight into the dynamics of the disordered regions of polypeptides can be gained through fitting of the ^15^N *R*_2_ relaxation rate data to models for polypeptide motion (Figure 7) [7], [33]. These models generally assume that the effect on the motion of an amino acid due to neighbouring residues decreases exponentially as the distance between them increases. According to Schwarzinger *et al.* [32], the sequence dependent *R*_2_ relaxation rates are given by the following equation:

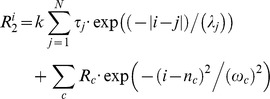


where the first term accounts for the intrinsic relaxation of the polypeptide and the second term describes additional interactions arising from local amino acid clusters. Moreover, 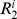 is the relaxation rate of residue 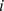, 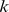 is an empirical scaling constant, N is the total number of residues, and 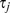 and 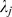 are the intrinsic correlation time of residue 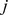 and the persistence length for segmental motion of the polypeptide, respectively. In this approach, the intrinsic correlation time of a given residue is taken to be proportional to 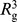, where 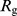 is its radius of gyration, and 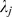 is assumed to be 1 for glycine and alanine, and 7 for all other residues. The clusters are then defined by an intrinsic *R*_2_ relaxation rate (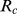), centered at residue 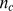 with a half-width of 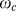. In the approach initially proposed by Klein-Seetharaman *et al.*, 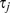 in the first term of Eqn. 1 is set at 1 so that all amino acid residues contribute uniformly to the calculated *R*_2_. Here, we follow the approach of Schwarzinger *et al.* because glycine and alanine allow greater backbone flexibility than other amino acids, suggesting that the amino acid size is a relevant factor. This effect may be particularly pronounced for small polypeptide chains as is the case for TC5b. However, the hydrophobic cluster identified above is very similar for both approaches (Figure 7). Confirmation that side chains of residues neighbouring the central cluster depicted in this representation are indeed relatively close to each other and have constrained independent motions is provided by the observation of an associated network of inter-residue ^1^H-^1^H NOEs existing between these side chains (Table 1). Chemical shift values that deviate significantly from primary values provide additional experimental support for the presence of these clusters (*vide supra*).

**Figure 8:**
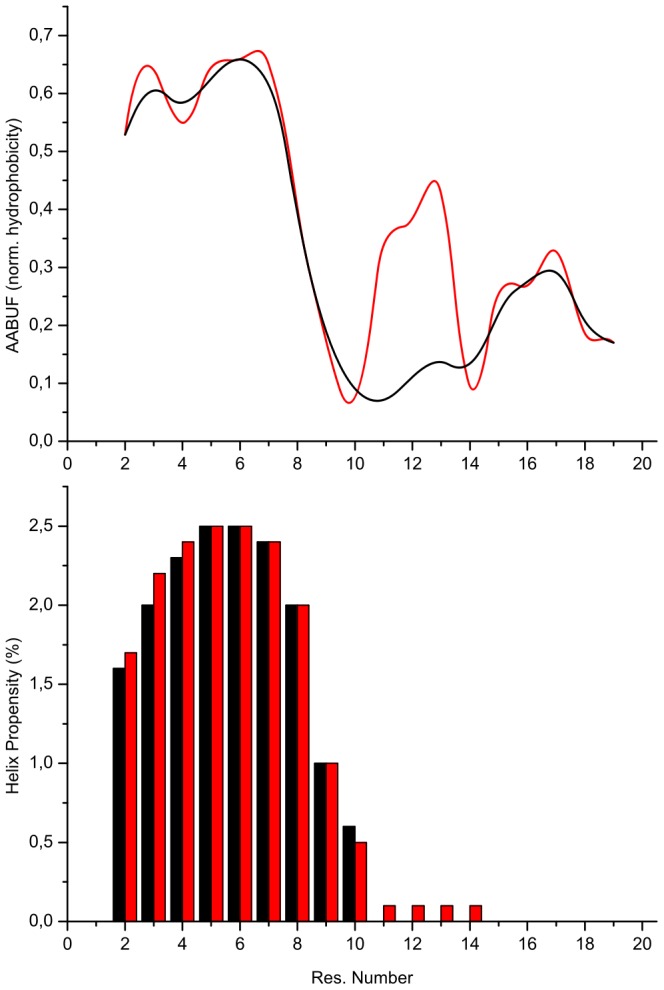
Average area buried upon folding & helix propensities. Plot of the average area buried upon folding (AABUF; upper part) and the helix propensities (lower part) for TC5b (black) and its P12W (red) point mutant.

The hydrophobic character of the cluster is shown by the nature of the residues with resonance chemical shifts significantly different from the primary values, which are predominantly non-polar in nature, and from the correlation between the clusters and the average area buried upon folding (AABUF) of the amino acid sequence (Figure 8). The AABUF is proportional to the hydrophobic contribution of a residue to the conformational free energy of a protein [45] and it has been shown to correlate with sequence-dependent dynamic variations due to cluster formation in, for example, urea-unfolded myoglobin [32] and the natively unfolded translocation domain of colicin E9 [34]. The AABUF profile for the TC5b amino acid sequence mirrors 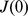 and, more interestingly, corresponds approximately to a region of high helical propensity predicted using the algorithm AGADIR [46]. The correspondence, which is not an exact one in terms of the sizes of the AABUF profile and AGADIR propensities but is clear in terms of the regions of the sequence highlighted by both methods, results from the presence of hydrophobic residues promoting both the formation of helices and the observed cluster in urea-denatured TC5b.

Taken together, the data suggest that, very similar to other proteins denatured at high concentrations of urea [5], [7], [32], [33], [47], [48], 6 M urea-unfolded TC5b lacks regular secondary structure, but is a dynamic ensemble of species that contain a distinct cluster of locally interacting residues, comprising both aromatic and aliphatic side chains.

### Urea-unfolded P12W-TC5b and W6G-TC5b

Native and/or non-native hydrophobic interactions formed at the very early stages of the folding process have been identified as crucial prerequisites required for the productive folding of globular proteins to occur in a sequence-directed manner [49], [50]. To determine whether the hydrophobic interactions found in the denatured state of TC5b have an influence on the ultrafast folding, we subsequently studied two variants of the TC5b mini-protein, namely P12W-TC5b, a construct known to exhibit a higher thermal melting point and diffusion-limited folding kinetics (*k*_f_) of ca. 1 per microsecond [25], [51], [52], and, in addition, W6G-TC5b.

Prior to measuring backbone *R*_2_ values, the *in silico* native state structure of P12W-TC5b, was verified following an NOE-restrained structure calculation involving a total number 270 unambiguous mid- and long-range NOE contacts and a set of 10 three-bond H^N^-H_α_ coupling constants (Figure S2; BMRB/SMSDep entry 21004): the ensemble of calculated and energy minimized native P12W-TC5b conformers (Table S2) exhibits all the characteristic features found in the ensemble of native TC5b structures in addition to a few detectable differences; e.g. the side chain of Pro 18 now covers one face of the Trp 6 residue, whereas in the wild-type peptide, the opposite side of the Trp residue is covered by Pro 12, now being replaced by a second tryptophan residue in the mutant with the planes of the two indole moieties arranged perpendicular to each other in a characteristic ‘edge-to-face’ pairwise fashion. This structural arrangement found in the interior of the mutant hydrophobic core, in turn, results in a further compaction of the globular structure of the mini-protein’s native fold.

The ^1^H-^15^N HSQC spectrum of P12W-TC5b shows that the protein is highly unfolded in 6 M urea indicated – as in the case of 6 M urea-denatured TC5b – by a much more reduced chemical shift dispersion in the ^1^H dimension of the spectrum, as well as the observation of exchange-broadened cross-peaks (not shown). Interestingly, the HSQC spectrum of the P12W mutant does not exhibit a second set of signals corresponding to those found for wild-type TC5b (*vide supra*), indicating that the presence of a second conformation observed in the case of 6 M urea-denatured TC5b, is caused by the isomerization of Pro 12. Resonance assignments for ^1^H-^15^N HSQC spectra of the urea-denatured variant were obtained using the NMR assignments for 6 M urea-denatured WT TC5b as a guide, with 600 MHz ^1^H-^1^H-^15^N-NOESY-HSQC spectra providing confirmatory data. Chemical shift differences between common residues of the variants and wild-type TC5b in 6 M urea were generally negligible, apart from those at the site of the amino acid substitutions or close to them and, for P12W-TC5b, at specific additional sites as a consequence of pronounced ring current shifts caused by the presence of the additional Trp residue.

Backbone ^15^N *R*_2_ relaxation rates values were determined for 16 residues of the P12W-TC5b mutant in 6 M urea (Table S3). The sequence variation of the *R*_2_ values shows a behaviour very similar to that of wild-type TC5b, indicating that the residual structure found in urea-denatured TC5b is largely unaffected by the substitution of proline with tryptophan. On the contrary, significantly elevated relaxation values for Trp 12 and neighbouring residues on the order of up to ca. 5.5 s^−1^ seem to suggest that additional non-covalent Trp-Trp interactions, e.g. π-π stacking, lead to a further compaction of the denatured-state hydrophobic core, as compared to WT TC5b, reflected by higher *R*_2_ rates in those regions of the sequence identified to be structurally affected by the presence of sequence-remote interactions in the case of 6 M urea-denatured TC5b (Figure 7). Interestingly, the observation of a further compaction in the case of unfolded P12W-TC5b was verified independently by determining hydrodynamic radii of both 6 M urea-unfolded WT TC5b and its P12W mutant using pulsed field gradient-assisted diffusion NMR spectroscopy [28] (Figure S3). The relaxation analysis of W6G-TC5b – a species lacking the Trp 6 side chain crucial for the formation of the ‘high temperature’ hydrophobic core – of TC5b, on the other hand, shows that it behaves like a statistical random coil void of any sequence-remote amino acid side chain interactions even in the absence of denaturant. This conclusion was drawn following the analysis of the 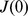 values being in the range between 0.6 and 3.2 s^−1^ along the entire sequence and exhibiting an almost perfect correlation between 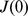 and the measured *R*_2_ values [37]. The lack of tertiary structure in native as well as chemically denatured ensembles of W6G-TC5b was further underscored by CD measurements, and, in addition, the absence of mid- or long-range proton-proton NOEs in spectra acquired at different mixing times between 250 and 500 ms; an observation further supported by MD-assisted structural studies on various TC5b single-site mutants [53]. As such, hydrophobic cluster formation seen in unfolded TC5b and P12W-TC5b seems to represent an important step during folding crucial not only for the formation of the native hydrophobic core but also for the formation of secondary structure elements, e.g. the alpha-helix found in the native state of TC5b and structurally similar analogues.

### Implications for TC5b Folding

Despite a plethora of structural information obtained *in silico* describing the folding behaviour of TC5b, only little experimental information is presently available dealing with the structural features of this highly unusual biomolecule. Moreover, most experiments carried out to obtain structural information on the conformational space sampled by the peptide on its way to the final, i.e. native, structure, concentrated on either the determination of folding kinetics or, alternatively, the structural features present under native conditions. For the first time, we show here on the level of individual amino acid residues that despite being significantly less ordered relative to the native state, 6 M urea-denatured TC5b exhibits persisting sequence-remote interactions involving a number of aliphatic amino acid side chains that form a distinct cluster of interacting residues. The existence of this structure was proven following the analysis of ^13^C secondary chemical shifts, NOE cross-peaks, and heteronuclear (^15^N) relaxation rates/NOE data complemented with a reduced spectral density treatment and cluster analyses of the latter. To a first approximation, this cluster corresponds to a region of the protein that forms an alpha-helix in the native state, although the centre of the cluster does not coincide perfectly with the centre of the native helix. This presumably results because hydrophobic helix-promoting residues also favour cluster formation in the unfolded state.

In addition, the structural analysis of two TC5b point mutants, P12W-TC5b and W6G-TC5b, revealed two further aspects of the folding features of wild-type TC5b: first, an increase of a tight side chain packing in the denatured state, as represented by enhanced Trp-Trp interactions in P12W-TC5b, leads to increased stability of the native state structure, indicated by both elevated melting temperatures *T*_m_ (42 vs. 56.9°C) and higher refolding rate constants *k*_f_ (4.1 vs. 0.94 per microsecond). Second, the elimination of *all* hydrophobic long- and mid-range side chain interactions found in the denatured state of TC5b (W6G-TC5b), does not only hinder the formation of a pre-existing globular cluster but, more importantly, also impedes the formation of secondary structure in all regions of the mini-protein.

The interplay between hydrophobic collapse and the formation of helical secondary structure in the early stages of protein folding has been widely discussed [54]–[62]. Clustering of hydrophobic groups to form a compact globule in the absence of hydrogen bond formation is energetically unfavorable [39]. In addition, regions corresponding to native helices are generally unable to form substantial persistent structure in the absence of additional stabilizing interactions, as has been demonstrated by studies of peptide fragments, including peptides structurally equivalent to TC5b. As a consequence, hydrophobic collapse and hydrogen bond formation occur jointly in the earliest stages of folding [63]–[65]. For TC5b, we suggest that these stages will involve coalescence, i.e. hydrophobic collapse [66], [67], of the non-polar residues as solvating urea is removed from the protein backbone, concomitant with the formation of inter-residue hydrogen bonds, at least some of which might involve native-like pairings. Further consolidation of the cluster will subsequently result in a compaction of the hydrophobic core, combined with the development of stable hydrogen bonded structure in the helix resulting in the population of the characteristic native alpha-helical peptide. This intricate interplay between globular cluster compaction and helix formation during folding of the peptide has lately been identified independently following phi-value analyses on different TC5b mutants [68].

The data presented here indicate that at least in some cases native *and* non-native interactions might be required from the start to ensure a rapid folding of the protein and that folding rates can be increased when more pronounced residual interactions are introduced. In another case, fluorescence studies on TC5b analogues have shown that a decrease in folding speed is found when elements of the residual structure in the denatured state are removed [27]. Simulation studies starting with an extended structure suggest that there is an initial collapse event, with different hydrophobic interactions formed depending on the specific trajectory chosen [11], [69]. In agreement with some of the cluster-forming interactions found *in silico* after only a few nanoseconds of MD simulation time, we find that specific native and non-native contacts already pre-exist in the unfolded ensemble and hence, it appears that the presence of hydrophobically collapsed structures in the equilibrated unfolded state may be a significant factor in accelerating folding. Moreover, the simple helix topology of TC5b, combined with the short length and polar nature of the interconnecting loop regions, dictates that clusters of non-random interactions will form in regions corresponding to the native helix. For proteins with more complex topologies, however, including those with long loops containing hydrophobic residues, or proteins comprised of multiple domains, the clusters may involve even more non-native interactions which have to be reorganized in order for the protein to fold to the native state.

Hence, even though a complete understanding of the folding mechanism of TC5b requires further work, e.g. analysis of the structural properties of TC5b at varying denaturant concentrations and the subsequent transition state indispensable for the formation of a putatively populated folding intermediate [35], [70], the results presented here provide new insights into the structure and dynamic properties of the 6 M urea-unfolded state revealing significant non-random structure within this ensemble from which folding might commence. As such, we believe that the data help to bridge the gap between both the experimental work performed on ultrafast folding proteins and, on the other hand, molecular dynamics simulation studies.

## Materials and Methods

Recombinant over-expression of all peptides was performed according to the following procedures: the cDNA coding sequence for all TC5b peptide analogues, i.e. WT TC5b, P12W-TC5b, and W6G-TC5b, was synthesised by GeneArt (Regensburg, Germany) and ligated into the Dral and Xhol restriction sites of the pET41a vector (Merck Chemicals Ltd., Nottingham, UK). The resulting expression plasmid pET41a-TC5b was used for transformation of *E. coli* strain BL21(DE3)pRil (Stratagene, La Jolla, CA). For the expression of uniformly ^13^C- and ^15^N-labelled peptide, cells were grown in ^13^C-glucose- and ^15^NH_4_Cl-containing minimal medium at 37°C until the OD reached approximately 0.7. Recombinant protein production was induced by the addition of IPTG to a final concentration of 1 mM and incubated for 12 hours at 28°C. Cells were harvested through centrifugation at 5000 rpm for 20 minutes, resuspended in 20 ml of ice-cold standard GST loading buffer per litre of the original bacterial culture, and lysed by passing through a microfluidic high pressure device (Microfluidics, Newton, MA) at 5.5 bar. Cell debris was removed through centrifugation at 15000 rpm for 30 minutes and 20 ml portions of the supernatant containing the soluble protein fraction were applied to a glutathione-sepharose column (Clontech, Mountain View, CA). For GST-Tag™ removal, recombinant enterokinase (rEK; Merck Chemicals Ltd., Nottingham, UK) was added and the reaction was incubated for 96 h at 37°C in dedicated rEK cleavage buffer (Merck Chemicals Ltd., Nottingham, UK). The peptide-containing supernatant was collected and dialysed against H_2_O overnight, concentrated, and loaded onto a HiLoad™ 16/60 Superdex™ 30 prep grade gel-filtration column (GE Healthcare, Waukesha, WI) equilibrated in 25 mM NaH_2_PO_4_/Na_2_HPO_4_ (pH 5.8) buffer for final purification. The final yield of pure peptide was approximately 0.18 mg (TC5b), 0.14 mg (P12W-TC5b), or 0.15 mg (W6G-TC5b), respectively, per litre of bacterial culture. The mass, sequence, and purity of all peptides was verified via electrospray ionisation (ESI) MS and NMR spectroscopic methods.

NMR spectra were acquired using four-channel Bruker AVANCE spectrometers equipped with cryogenic (CPCQI) or non-cryogenic (QXI) 5 mm pulsed field gradient (PFG) probes operating at ^1^H frequencies of 599.92 or 600.25 MHz, and ^15^N frequencies of 60.25 or 60.82 MHz, respectively. Standard two- and three-dimensional pulse sequences used for the acquisition of all spectra were taken from the Bruker pulse sequence library and edited to satisfy sample-specific needs where required. ^1^H resonance assignments were extracted via analysis of homonuclear proton-proton TOCSY and NOESY spectra acquired with a spin-lock time of 80 ms (TOCSY) and mixing times of 150 or 400 ms, respectively (NOESY). The number of complex points in the direct dimension corresponded to 2048 while the indirect dimension contained between 256 and 512 complex points at a sweep width of approximately 12 ppm. All spectra were processed using NMRPipe [71] and/or TopSpin™ 2.1 (Bruker BioSpin, Rheinstetten, Germany). Assignment and further interpretation of all spectra was performed using standard methods. The CARA software package was used to visualize, assign, and, in the case of the native TC5b-P12W mutant, integrate the spectra. Before Fourier transformation, a 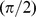-shifted squared sine-bell window function was applied to each dimension for apodization. Both dimensions were then zero-filled to round up the number of data points to the nearest power of two. ^1^H chemical shifts were referenced directly or, in the case of ^13^C and ^15^N chemical shifts, indirectly to 2,2-dimethyl-2-silapentene-5-sulfonate sodium salt (DSS) [72]. All NMR samples contained the respective peptide (1 mM) dissolved in 25 mM sodium phosphate buffer, a H_2_O/D_2_O ratio of 9/1, and 0.1 mM DSS for internal referencing. Sample solutions containing the native peptide were neutral (pH ∼ 6.8) whereas the pH value of the 6 M urea-containing solutions was 3.5. All pH values are direct readings and have not been corrected for the deuterium isotope effect. NMR spectra of the native state were recorded at a temperature of 5°C where, according to TC5b’s temperature denaturation curve acquired in the absence of urea, almost all – i.e. more than 95 per cent – protein molecules are fully folded [9]. 6 M urea-denatured protein samples, on the other hand, were observed at a temperature of 15°C, thereby ensuring the almost exclusive presence, i.e. more than 98%, of unfolded protein fraction in the sample tube according to TC5b’s urea denaturation curve described in the literature [28] and verified in our own laboratory following both CD and fluorescence excitation measurements at varying denaturant concentrations between 0 and 8 M urea conducted in steps of 0.5 M urea.

Chemical shift differences were calculated using the CSI module of NMRView [73], taking into account the sequence variation of the primary chemical shift values, using the 8 M urea database at pH 2.5. The chemical shifts recorded for glutamate and aspartate residues were significantly different from the primary values, due to the difference in pH between the reference data and the measured chemical shifts of urea-unfolded TC5b, and were not taken into account in the present analysis.

Heteronuclear ^13^C NMR experiments were acquired with a sweep width of 12.2 ppm in the direct (^1^H) dimension, and 80 ppm in the indirect, i.e. ^13^C, dimension at 1024*500 points of digital resolution. Moreover, ^15^N NMR experiments were recorded with a sweep width of 12.2. ppm in the direct (^1^H) dimension, and in the ^15^N dimension the sweep width was either 30 ppm centered around 115 ppm in the case of the native peptide and 26 ppm centered around 120 ppm for 6 M urea-denatured peptide: ^1^H-^15^N HSQC spectra were acquired at ^15^N frequencies of 60.25 MHz and 2048*512 points. HNHA spectra were acquired with 2048 and 128 complex points in the direct and indirect ^1^H dimensions and 32 complex points in the ^15^N dimension. Backbone ^15^N longitudinal (*R*_1_) and transverse (*R*_2_) relaxation rates and the heteronuclear NOE were measured as described in the literature [36], [74], [75] using sensitivity enhancement in the back transfer [76]. All spectra were acquired with 2048 points in the direct dimension (^15^N) and 128 complex points in the indirect dimension (^15^N). For the measurement of *R*_1_ and *R*_2_ relaxation rates, eight transients each were recorded using ten different mixing times between 10 and 1200 ms (*R*_1_) and 15.6 and 469.4 ms (*R*_2_), respectively. For two mixing times in each series, measurements were repeated to estimate the experimental error. Two experiments were recorded in an interleaved manner for the measurement of the heteronuclear NOE, one with proton presaturation – applied by a series of 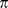 high power proton pulses during the recycle delay – and one without proton presaturation. The recycle delay (RD) was 6 s for the heteronuclear NOE experiment and 3 s for *R*_1_ and *R*_2_ experiments. Processing of the relaxation data and measurement of peak heights was carried out using NMRPipe. Data were Fourier transformed applying zerofilling and apodization by a 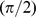-shifted squared sine-bell window function; only the ^1^H^N^ region of the spectra was retained. *R*_1_ and *R*_2_ relaxation rates were fitted as single exponential decays according to: 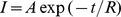 with 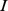 being the peak height data, 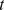 being the relaxation delay, 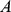 the amplitude, and *R* either *R*_1_ or *R*_2_ [77], [78]. All data fitting was carried out using the Origin 8.5 software package (OriginLab, Northampton, MA). The heteronuclear NOE was determined by dividing the peak heights in the spectra without presaturation by the ones with presaturation. Reduced spectral density mapping was performed according to procedures described in the literature [42], [43]. The spectral density functions 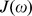, 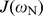, and 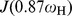 were obtained assuming that the variation in 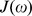 is relatively constant between 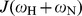 and 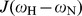. Chemical shift anisotropy was taken to be 172 ppm and the NH bond length was 1.02 Å. Helix propensities (%) were calculated with AGADIR [46]. AABUF values were determined using the Expert Protein Analysis System (ExPASy) [79] tool ProtScale and normalized between 0 and 1. All structural figures in this report were prepared using the software MOLMOL [80]. The structural coordinates of the native TC5b-P12W mutant including the complete ^1^H chemical shift assignments acquired in neutral aqueous buffer have been deposited (BMRB/SMSDep entry 21004).

## Supporting Information

Figure S1 **Comparison of ^13^C secondary chemical shifts for 6 M urea-denatured TC5b.** Shown are the calculated ^13^C secondary chemical shifts for 6 M urea-denatured TC5b using two different sets of random coil chemical shift values from Schwarzinger et al. (red) and Mulder and co-workers (black), respectively. The difference of the calculated values between the two sets is, in most cases, negligible.
(TIF)

Figure S2 **Stereo view backbone representation of native P12W-TC5b.** Shown is the ensemble of the twenty best structures (heavy atom r.m.s.d. 0.56±0.15 Å) calculated from ^1^H NMR data acquired in neutral aqueous buffer. All side chain nuclei are displayed for Tyr 3 (orange), Ile 4 (light green), Trp 6 (magenta), Leu 7 (cyan), Trp 12 (red), Pro 17 (black), Pro 18 (green), and Pro 19 (blue).
(TIF)

Figure S3 **Hydrodynamic radii of 6 M urea-denatured TC5b and P12W-TC5b.** Shown are the diffusion profiles (relative signal intensity vs. applied field gradient strength) for both native and denatured forms of TC5b and P12W-TC5b. The diffusion coefficients derived from these data correspond to hydrodynamic radii (*R*_H_) of 9.92±0.15 Å and 9.28±0.1 Å for the denatured forms of TC5b and P12W-TC5b, respectively.
(TIF)

Table S1 **Scalar H^N^H_α_ coupling constants [Hz] for native & 6 M urea-denatured TC5b.**
(DOC)

Table S2 **NMR structure statistics.**
(DOC)

Table S3 **Observed, calculated, and intrinsic** ***R*** **_2_ Relaxation Data for 6 M urea-denatured P12W-TC5b (c.f.** **Fig. 6** **).**
(DOC)
