# Supplementary figures and images for: Atomic-Level Structure Characterization of an Ultrafast Folding Mini-Protein Denatured State
Source: PLoS One. 2012 Jul 27;7(7):e41301. doi: 10.1371/journal.pone.0041301 (PMC3407199; doi:10.1371/journal.pone.0041301)

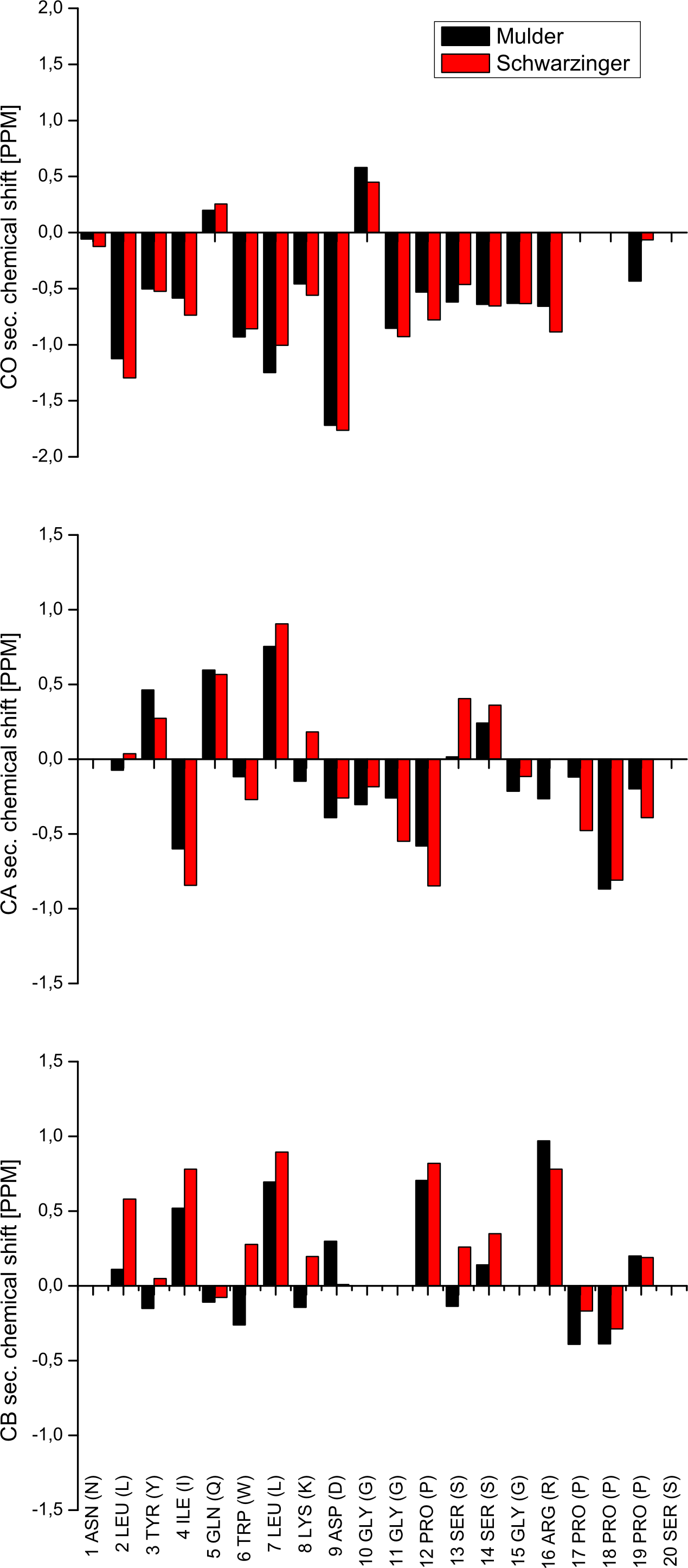

Supplement: Figure S1 — Comparison of 13C secondary chemical shifts for 6 M urea-denatured TC5b. Shown are the calculated 13C secondary chemical shifts for 6 M urea-denatured TC5b using two different sets of random coil chemical shift values from Schwarzinger et al. (red) and Mulder and co-workers (black), respectively. The difference of the calculated values between the two sets is, in most cases, negligible. (TIF) [file pone.0041301.s001.tif]

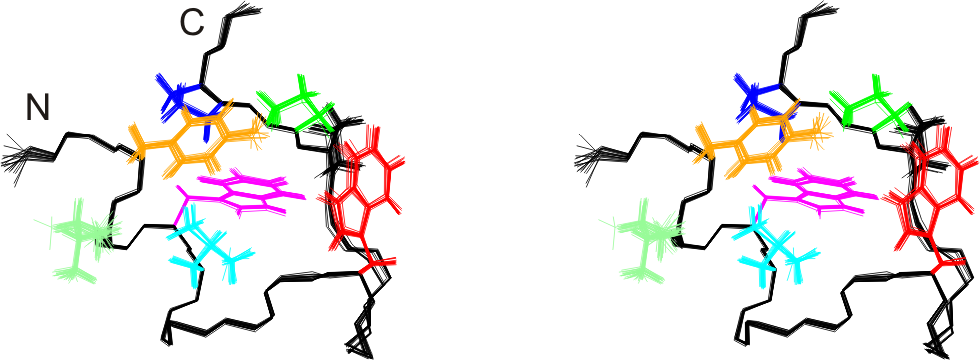

Supplement: Figure S2 — Stereo view backbone representation of native P12W-TC5b. Shown is the ensemble of the twenty best structures (heavy atom r.m.s.d. 0.56±0.15 Å) calculated from 1H NMR data acquired in neutral aqueous buffer. All side chain nuclei are displayed for Tyr 3 (orange), Ile 4 (light green), Trp 6 (magenta), Leu 7 (cyan), Trp 12 (red), Pro 17 (black), Pro 18 (green), and Pro 19 (blue). (TIF) [file pone.0041301.s002.tif]

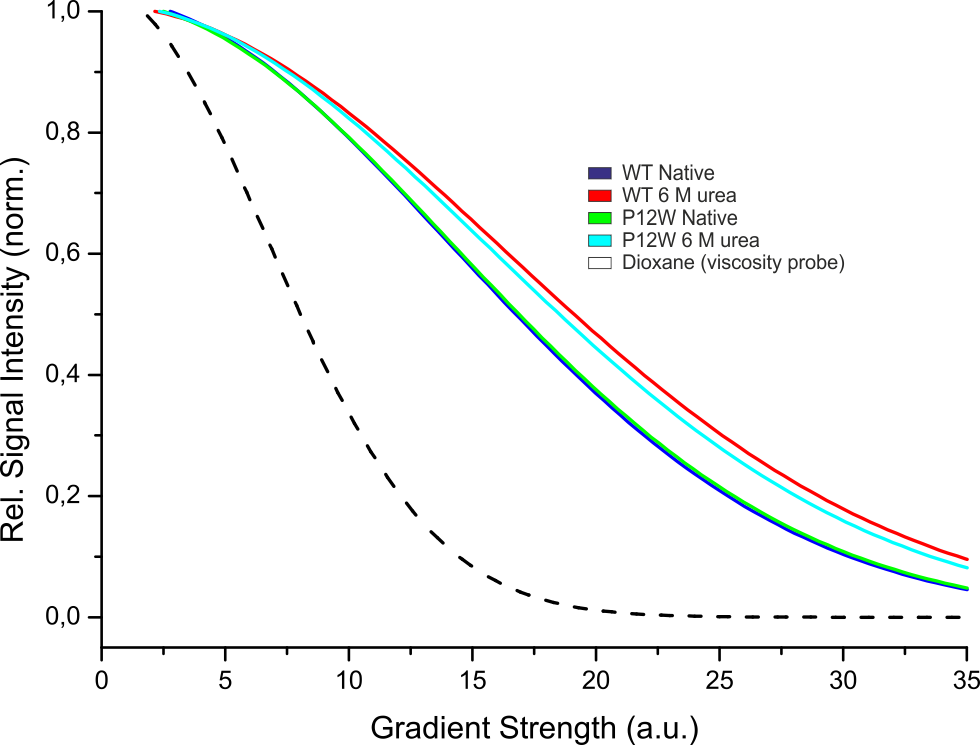

Supplement: Figure S3 — Hydrodynamic radii of 6 M urea-denatured TC5b and P12W-TC5b. Shown are the diffusion profiles (relative signal intensity vs. applied field gradient strength) for both native and denatured forms of TC5b and P12W-TC5b. The diffusion coefficients derived from these data correspond to hydrodynamic radii (R H) of 9.92±0.15 Å and 9.28±0.1 Å for the denatured forms of TC5b and P12W-TC5b, respectively. (TIF) [file pone.0041301.s003.tif]
