# Supplementary material for: Atomic-Level Structure Characterization of an Ultrafast Folding Mini-Protein Denatured State
Source: PLoS One. 2012 Jul 27;7(7):e41301. doi: 10.1371/journal.pone.0041301 (PMC3407199; doi:10.1371/journal.pone.0041301)
Supplement: Table S1 — Scalar HNHα coupling constants [Hz] for native & 6 M urea-denatured TC5b. (DOC) [file pone.0041301.s004.doc]

**Table S1. Scalar H^N^H_α_ coupling constants [Hz] for native & 6 M urea-denatured TC5b.**

| Residue No. | Residue | H^N^H_α_ coupling constant (native) [s^-1^] | H^N^H_α_ coupling constant (6 M urea) [s^-1^] |
| --- | --- | --- | --- |
|  |  |  |  |
| 1. | Asn | - | - |
| 2. | Leu | 5.54 | 5.97 |
| 3. | Tyr | 5.49 | 6.24 |
| 4. | Ile | 5.24 | 6.47 |
| 5. | Gln | 4.63 | 5.58 |
| 6. | Trp | 4.52 | 6.32 |
| 7. | Leu | 3.45 | 6.02 |
| 8. | Lys | 4.24 | 5.54 |
| 9. | Asp | 6.46 | 6.61 |
| 10. | Gly | - | - |
| 11. | Gly | - | - |
| 12. | Pro | - | - |
| 13. | Ser | 5.96 | 6.25 |
| 14. | Ser | 5.02 | 6.38 |
| 15. | Gly | - | - |
| 16. | Arg | 5.70 | 6.31 |
| 17. | Pro | - | - |
| 18. | Pro | - | - |
| 19. | Pro | - | - |
| 20. | Ser | 7.77 | 7.49 |
