# Supplementary material for: Atomic-Level Structure Characterization of an Ultrafast Folding Mini-Protein Denatured State
Source: PLoS One. 2012 Jul 27;7(7):e41301. doi: 10.1371/journal.pone.0041301 (PMC3407199; doi:10.1371/journal.pone.0041301)
Supplement: Table S2 — NMR structure statistics. (DOC) [file pone.0041301.s005.doc]

Table S2. NMR Structure Statistics.

| **Restraints** | | |
| --- | --- | --- |
|  | |  |
| Type of restraint: | |  |
|  |  |  |
| Distance restraints: | Type of Restraint: | Number: |
|  | Intraresidue | 88 |
|  | Sequential | 80 |
|  | $i,i+n,\text{ }n=2,3,\text{or }4$ | 48 |
|  | $i,i+n,\text{ }n\geq5$ | 54 |
| Scalar H^N^H_α_ coupling constants: | | 10 |
| **Structure statistics** | | |
|  | |  |
| E_LJ_ (kcal mol^-1^) | | $0.5\pm0.02$ |
|  | Type of restraint: |  |
| RMS restraint violations: | Upper distance (Å) | $0.0041\pm0.0011$ |
|  | Lower distance (Å) | $0.0001\pm0.0002$ |
|  | van-der-Waals (Å) | $0.7\pm0.0$ |
|  | Scalar couplings (Hz) | $0.12\pm0.01$ |
| Max. distance violation (Å) | | 0.15 |
| Max. scalar coupling violation (Hz) | | 0.12 |
|  |  |  |
| Ramachandran angles (%) | Most favoured | 100 |
|  | Additional allowed | 0 |
|  | Generously allowed | 0 |
|  | Disallowed | 0 |
|  | | |
| **Convergence within final ensemble, atomic r.m.s. deviations (Å)** | | |
|  |  |  |
| RMSD (Å) | Backbone | $0.15\pm0.05$ |
|  | Heavy atom | $0.56\pm0.15$ |
