# Supplementary material for: Atomic-Level Structure Characterization of an Ultrafast Folding Mini-Protein Denatured State
Source: PLoS One. 2012 Jul 27;7(7):e41301. doi: 10.1371/journal.pone.0041301 (PMC3407199; doi:10.1371/journal.pone.0041301)
Supplement: Table S3 — Observed, calculated, and intrinsic R 2 Relaxation Data for 6 M urea-denatured P12W-TC5b (c.f. Fig. 6 ). (DOC) [file pone.0041301.s006.doc]

**Table S3. Observed, calculated, and intrinsic *R*_2_ Relaxation Data for 6 M urea-denatured P12W-TC5b (c.f. Fig. 7).**

| Residue No. | Residue | *R*_2_ observed [s^-1^] | Err. *R*_2_ obs. [s^-1^] | *R*_2_ calc. [s^-1^] | *R*_2_ intrinsic [s^-1^] |
| --- | --- | --- | --- | --- | --- |
|  |  |  |  |  |  |
| 1. | Asn | - | - | 1.80577 | 1.80575 |
| 2. | Leu | 2.04273 | 0.01587 | 2.0483 | 2.03919 |
| 3. | Tyr | 2.60383 | 0.02187 | 2.7588 | 2.26178 |
| 4. | Ile | 6.04028 | 0.08672 | 6.08293 | 2.3916 |
| 5. | Gln | 6.74429 | 0.04774 | 6.43849 | 2.51568 |
| 6. | Trp | 3.5783 | 0.02031 | 4.09337 | 2.61278 |
| 7. | Leu | 4.24325 | 0.04033 | 4.15309 | 2.608 |
| 8. | Lys | 3.496 | 0.01229 | 3.59055 | 2.60401 |
| 9. | Asp | 3.59545 | 0.03515 | 2.82117 | 2.52379 |
| 10. | Gly | 3.1409 | 0.06093 | 2.82987 | 2.4531 |
| 11. | Gly | 3.50856 | 0.05314 | 3.75642 | 2.43256 |
| 12. | Trp | 5.4946 | 0.06238 | 5.12453 | 2.70074 |
| 13. | Ser | 4.74268 | 0.03046 | 3.74878 | 2.55617 |
| 14. | Ser | 2.09317 | 0.03341 | 2.77071 | 2.42207 |
| 15. | Gly | 1.94801 | 0.05706 | 2.48715 | 2.44937 |
| 16. | Arg | 3.06982 | 0.01519 | 2.52842 | 2.52674 |
| 17. | Pro | - | - | 2.46186 | 2.46183 |
| 18. | Pro | - | - | 2.33215 | 2.33215 |
| 19. | Pro | - | - | 2.13508 | 2.13508 |
| 20. | Ser | 1.64653 | 0.01185 | 1.86656 | 1.86656 |
